# The Predictive Role of Thyroid Hormone Levels for Early Diabetic Retinal Changes in Experimental Rat and Human Diabetes

**DOI:** 10.1167/iovs.62.6.20

**Published:** 2021-05-19

**Authors:** Anna Énzsöly, Rozina I. Hajdú, Zsolt Turóczi, Irén Szalai, Erika Tátrai, Fanni Pálya, Zoltán Z. Nagy, Csaba Mátyás, Attila Oláh, Tamás Radovits, Klaudia Szabó, Bulcsú Dékány, Arnold Szabó, Ákos Kusnyerik, Petra Soltész, Dániel S. Veres, Anikó Somogyi, Gábor M. Somfai, Ákos Lukáts

**Affiliations:** 1 Department of Ophthalmology, Semmelweis University, Budapest, Hungary; 2 Department of Anatomy, Histology and Embryology, Semmelweis University, Budapest, Hungary; 3 2nd Department of Internal Medicine, Semmelweis University, Budapest, Hungary; 4 Heart and Vascular Center, Semmelweis University, Budapest, Hungary; 5 Department of Biophysics and Radiation Biology, Semmelweis University, Budapest, Hungary; 6 Eye Clinic, Stadtspital Waid and Triemli, Zürich, Switzerland; 7 Werner H. Spross Foundation for the Advancement of Research and Teaching in Ophthalmology, Zürich, Switzerland

**Keywords:** thyroid hormones, diabetic retinopathy, color vision, opsin expression, cones

## Abstract

**Purpose:**

In diabetic subjects, early visual functional alterations such as color vision deficiencies (CVDs) are known to precede clinically apparent diabetic retinopathy. Prominent photoreceptor outer segment degeneration and an increase in the number of retinal dual cones (co-expressing S- and M-opsins simultaneously) have been described in diabetic rat models, suggesting a connection with the development of CVDs. As cone opsin expression is controlled by thyroid hormones, we investigated the diabetic retina in association with thyroid hormone alterations.

**Methods:**

In rat models of type 1 and 2 diabetes, dual cones were labeled by immunohistochemistry, and their numbers were analyzed in relation to free triiodothyronine (fT3) and free thyroxine (fT4) levels. Quantification of dual cones was also performed in human postmortem retinas. Additionally, a cross-sectional case–control study was performed where thyroid hormone levels were measured and color vision was assessed with Lanthony desaturated D15 discs.

**Results:**

A higher number of dual cones was detectable in diabetic rats, correlating with fT4 levels. Dual cones were also present in postmortem human retinas, with higher numbers in the three diabetic retinas. As expected, age was strongly associated with CVDs in human patients, and the presence of diabetes also increased the risk. However, the current study failed to detect any effect of thyroid hormones on the development of CVDs.

**Conclusions:**

Our results point toward the involvement of thyroid homeostasis in the opsin expression changes in diabetic rats and human samples. The evaluation of the possible clinical consequences warrants further research.

The clinical classification of retinopathy and its therapy are all based on the funduscopically detectable vascular pathology.^1^ However, multiple clinical studies have demonstrated neuroretinal structural changes,^2^^–^^4^ electroretinographic abnormalities,^5^ and color vision deficiencies (CVDs) preceding clinically evident vasculopathy.^6^^–^^9^ Understanding the underlying pathology of functional visual alterations may help us to find prognostic markers for the development of future vasculopathy.^7^^,^^10^^,^^11^ Histological studies have already been performed by us and others on different animal models.^12^^–^^17^

As far as the photoreceptors were concerned, type 1 and 2 diabetic models showed a peculiar similarity. The outer segments of rods and middle-wavelength sensitive cones (M-cones) showed a severely deformed morphology, and a special shift in cone opsin expression pattern was detectable, resulting in an increase in the number of dual cones that simultaneously express short-wavelength sensitive- (S-) and M-opsins.^18^^,^^19^ In murine models, Lyubarsky and co-workers^20^ applied electroretinography to show that when M-opsin is expressed to some degree in the S-cones, the responsiveness of the S-cone–driven signaling pathway can be completely suppressed by an orange conditioning flash. Therefore, color discrimination ability is injured in dual cones. It is currently unknown whether dual cones are also present in human diabetic patients and whether their presence can be connected to the CVD detected.

The mechanisms involved in the prominent increase in the number of dual cones in diabetes are still unclear, presenting a challenging question. Dual elements are common constituents of the developing rat retina, representing a transitional stage of M-cone development. All cones initially express only S-opsin. Later, most cones switch on M-opsin and switch off S-opsin expression, transdifferentiating into definitive M-cones.^21^ For a short interval, these cones contain both types of pigments and are visible as dual elements. The minority of the cones do not undergo this opsin switch and thus remain definitive S-cones. The above process is controlled by thyroid hormones acting through thyroid hormone receptor β2. A homodimer form is required to activate M-opsin expression, and a heterodimer with retinoid X receptor-γ inactivates S-opsin expression.^22^^,^^23^ The process seems to be reversible, as inadequate thyroid hormone levels may lead to the reappearance of dual cones or an increase in the number of S-cones, even in adult rats.^24^

Taking all this into account, it is possible that changes in thyroid homeostasis may be responsible for the appearance of dual cones. It is well documented that diabetes often causes fluctuations in thyroid hormone levels (both elevated and reduced levels occur more frequently than in people without diabetes),^25^^,^^26^ thus this proposition seems to be logical.

Even in euthyroid patients, diabetes appears to affect thyroid function first at the level of hypothalamic control and then in peripheral tissues, as well. In experimentally induced diabetes, thyroid-stimulating hormone (TSH) release is reduced along with a decrease in peripheral deiodinase activity, and as such a defect in the conversion of thyroxin to triiodothyronine (T4 to T3) has been reported.^27^ Patients with poorly controlled diabetes may have an impaired TSH response to thyrotropin-releasing hormone stimulation with decreased T4-to-T3 conversion. A significant improvement in the above changes by optimizing glycemic control has also been reported.^28^

Furthermore, circulating thyroid hormones have an impact on the regulation of glucose homeostasis.^28^ T3 directly increases pancreatic beta cell activity and controls insulin secretion and intracellular glucose availability. Subtle changes in the levels of serum thyroid hormones, even within the physiological range, can induce insulin resistance or diabetes. Individual changes in TSH and thyroid hormone levels within the normal reference range represent an additional risk factor of incident type 2 diabetes. Thyroid disorders, as well as diabetes, are frequently subclinical with no overt symptoms and thus are often undiagnosed and untreated.^29^ The aim of the present study was to investigate the possible connection between the thyroid hormones and diabetic neuroretinal alterations in animal experiments, postmortem human histology, and a pilot clinical study.

## Methods

### Animal Models

All procedures were performed in concordance with the ARVO Statement for the Use of Animals in Ophthalmic and Vision Research and were approved by the Ethics Committee of Semmelweis University and by the Animal Health and Animal Welfare Directorate of the National Food Chain Safety Office (22.1/1162/3/2010). Animals were housed individually in a room with constant temperature (22°C ± 2°C) under a 12-hour/12-hour alternating light/dark cycle.

Samples from two different types of rat models of diabetes were used. Zucker diabetic fatty (ZDF) inbred male rats (*n* = 7 for diabetic and *n* = 8 for control), purchased at 6 weeks of age (Charles River Laboratories, Sulzfeld, Germany), served as type 2 diabetes models. Due to a genetic mutation of the leptin receptor gene, homozygous recessive (fa/fa) ZDF rats develop fasting hyperglycemia, obesity, and type 2 diabetes under a special diet. As homozygous dominant (+/+) and heterozygous (fa/+) genotypes remain normoglycemic (ZDF lean), those were used as control animals in the experiment. Animals were fed a special diet (Purina 5008) and water ad libitum. Body weights and blood glucose levels (Accu-Chek Sensor; Roche, Mannheim, Germany) were checked regularly throughout the observation period, as described earlier elsewhere.^14^ Experimental procedures were performed at the age of 32 weeks following 25 weeks of diabetes duration.

To mimic type 1 diabetes, streptozotocin (STZ, 60 mg/kg; Sigma-Aldrich Kft., Budapest, Hungary) was injected intraperitoneally into 8-week-old male Sprague Dawley rats (*n* = 8 in the diabetic and *n* = 5 in the control group; Charles River Laboratories). Control animals were treated with an equivalent amount of buffer only (citrate buffer, pH = 4.5; 0.1 mol/L). Seventy-two hours after the injection, blood glucose concentration was measured with a digital blood glucose meter (Roche Accu-Chek Sensor). Animals with a blood glucose level > 15 mmol/L were considered diabetic and included in the study. Animals received a standard laboratory rat diet and water ad libitum throughout the study. Experiments were performed after 8 weeks of diabetes duration. Data for the animals and control of diabetes are given in detail elsewhere.^14^^,^^30^

### Tissue Preparation for Immunohistochemistry

At the end of the observation period, serum samples were collected, and the retinas were fixed as described elsewhere.^14^ In brief, animals were anesthetized with 1% to 2% isoflurane in 100% oxygen and placed on a heating pad (body temperature was maintained at 37°C). To remove erythrocytes from tissues, an in vivo perfusion was performed with a total volume of 40 mL oxygenated Ringer's solution (37°C, 8 mL/min), and the animals were decapitated. The eyes were oriented and removed. The cornea, the lens, and the vitreous body were dissected, and the remaining eyecup was placed into fixative (4% paraformaldehyde [PFA] diluted in 0.1-M phosphate buffer (PB; pH 7.4) for 2 hours at room temperature. After rinsing, the retinas were carefully detached with the orientation preserved and treated further as whole mounts. In ZDF rats, eyecups were prepared for cryosectioning as already detailed elsewhere,^14^ and 20-µm-thick cryosections were cut vertically and stored at −20°C until use.

### Postmortem Human Retinal Tissue Samples

The work followed the tenets of the Declaration of Helsinki and was approved by the Regional and Institutional Scientific and Research Ethics Committee of Semmelweis University (TUKEB 242/2015). Anonymized postmortem human retinal tissues (from eight control and three diabetic subjects) were obtained from enucleated eyes donated for cornea transplantation. Data of donors are summarized in Table 1.

**Table 1.** Data for the Human Retina Donors Used in the Study

Donor Identifier	Age/Sex	Postmortem Time	Known Pathology
C1	61 y/M	<90 min	Died from cerebral hemorrhage, chronic pancreatitis
C2	62 y/M	<90 min	Died from brainstem infarction
C3	20 y/M	<60 min	Died from subarachnoid bleeding
C4	54 y/M	∼3 min	Died from subdural bleeding
C5	43 y/M	∼90 min	Died from subdural bleeding, alcohol abuse
C6	53 y/F	<90 min	Died from subarachnoid bleeding
C7	62 y/F	<90 min	Died from subarachnoid bleeding
C8	58 y/M	<90 min	Died from cerebral hemorrhage, severe hypertension
Dm1	71 y/M	∼200 min	Died from cardiac failure, type 2 diabetes (for 30 y, 10 y insulin therapy), nonproliferative retinopathy, hypertension
Dm2	69 y/M	<90 min	Died from cardiac failure, type 2 diabetes (for 1 y)
Dm3	56 y/F	Within 7 min	Died from brainstem hematoma, cerebellar tonsil herniation, type 2 diabetes (for less than 10 y, insulin-treated), hypertension, diabetic nephropathy, diabetic and hypertonic retinopathy, maculopathy, hypothyroidism, several intraretinal bleedings in the retina, scars of laser photocoagulations

Note that all diabetic patients had type 2 diabetes, and the patient identified as Dm3 also had hypothyroidism listed in her medical history. Identifiers C1 to C8 belong to the data of non-diabetic donors (*n* = 8) and the data for the diabetic donors are labeled as Dm1 to Dm3 (*n* = 3). M, male; F, female.

After the corneas were harvested, the eyecups were cut into pieces with preserved orientation. The temporal retinal piece was dissected from the choroid and the pigment epithelium and then fixed in ample 4% PFA for 2 hours at room temperature. After extensive rinsing in 0.1-M PB, samples were further processed for immunohistochemistry as described below. In this study, only far peripheral pieces from the temporal retinal part were used, including the ora serrata.

### Immunohistochemistry and Imaging

In Sprague Dawley rats, cone subtypes were visualized by immunohistochemistry using a protocol already published, with some modifications.^12^^,^^14^ Non-specific binding was blocked with a 1% solution of bovine serum albumin (in 0.1-M PBS, pH 7.4) overnight at 4°C, with 0.4% Triton X-100 (Sigma-Aldrich Kft.) added. This solution was also used to dilute primary antibodies. Unlike in previous reports, for the present study primary antibodies were applied for 7 days (4°C) using continuous agitation. S-opsin content was labeled with a monoclonal antibody, OS-2, diluted 1:5000 (produced in the laboratory of Ágoston Szél and Pál Röhlich^18^), and M-opsin was stained with a commercially available AB5405 (1:1000, Anti-Opsin Antibody, Red/Green, produced in rabbits; MilliporeSigma, Billerica, MA, USA).^31^ The bound primary antibodies were visualized by species-specific fluorescent dyes (Alexa 488 and Alexa 594 conjugates, 1:200; Thermo Fisher Scientific, Waltham, MA, USA) applied for 24 hours at room temperature with continuous agitation. Human retinal pieces were treated similarly, except for the secondary antibodies, which were incubated for 48 hours.

In the case of ZDF rats, due to the small number of specimens available, labeling of cones was carried out on cryosections only. The primary antibodies were applied for only 24 hours. Detailed protocols for the sectioning and staining of ZDF rats have been published elsewhere.^14^ Representative images were taken with a Zeiss LSM 780 Confocal System coupled to a Zeiss Axio Imager upright microscope using a 40× immersion objective and Zen 2012 software (Carl Zeiss Meditec AG, Oberkochen, Germany).

### Quantification of Cones

Inspection of the retinas and quantification of cones were performed with a Zeiss Axiophot microscope using a 40× objective and the appropriate filter sets. Samples from rat retinal flatmounts were taken from three positions. The mean value for 12 evenly distributed areas (three per quadrant), 77,175 µm^2^ each, located immediately at the ora serrata, gave the extreme peripheral values for dual cones. Equal areas shifted 200 µm toward the center were used to obtain the peripheral values. Central values were derived from four centrally located counting frames (one in each quadrant) of 77,175 µm^2^ each. Additionally, mean values for the number of S- and M-opsin positive cones, dual cones, and total cone number were also calculated from the central counting frames. The ratio of S-cones to M-cones and the ratio of dual cones to total cone number were also estimated. In human flatmounts, dual cones were counted in the retinal width of 436 µm along the ora serrata. The number of S- and M-opsin–positive cones and total cone number were analyzed in three counting frames per each retina, 22,801 µm^2^ each. The ratio of S-cones to M-cones and the ratio of dual cones to total cone number were estimated and presented from these results for each donor retina.

### Quantification of Thyroid Hormone Levels (TSH, fT3, fT4)

The quantifications were performed using chemiluminescent microparticle immunoassays, including the Architect TSH reagent kit (B7K62H), Architect free triiodothyronine (fT3) assay (B7K63H), and Architect free thyroxine (fT4) assay (B7K65H; Abbott Ireland, Longford, Ireland), with an Architect i2000SR Immunoassay Analyzer following the recommendations of the supplier. In the case of animal samples, only fT3 and fT4 levels were evaluated.

### Human Subjects

We performed a preliminary cross-sectional case–control study, approved by the Regional and Institutional Scientific and Research Ethics Committee of Semmelweis University (TUKEB 242/2015). All subjects signed an informed consent form prior to the examinations, all of which followed the tenets of the Declaration of Helsinki. Patients with type 2 diabetes with a disease duration of less than 10 years and age-matched controls (with no history of diabetes mellitus) underwent a comprehensive physical and ophthalmological examination. Patients with any history of ocular pathologies (including funduscopically manifest diabetic retinopathy, ocular surgery in history, and congenital CVDs manifest on an Ishihara test), thyroid disease, or any treatment in the last 6 months with the potential to alter thyroid function (e.g., steroid treatment, chemotherapy, intensive care) were excluded.

Fasting blood samples were collected and transferred for laboratory testing using the current standardized laboratory guidelines. In addition to routine laboratory tests, TSH, fT3, fT4 levels, and fasting blood glucose levels were measured. In diabetic patients, hemoglobin A1c (HbA1c) levels were examined to assess glycemic control. All patients had a best-corrected visual acuity of 20/20 as assessed using the Early Treatment Diabetic Retinopathy Study (ETDRS) standardized testing chart.

Color vision was assessed using Lanthony desaturated D15 discs.^32^ All tests were performed under the same illumination conditions in the same environment, monocularly, using the full near correction of the patient. Manifest CVD and No CVD subgroups were created based on the number and magnitude of errors made in lining up the 15 desaturated discs. Manifest CVD was defined as at least six discs being skipped at least twice by the patient using the same eye. When the number of errors was less than this, the patient was assigned to the No CVD subgroup.

Finally, fundoscopy with a Volk +90D lens (Volk, Mentor, OH, USA) using a slit-lamp biomicroscope was carried out through a dilated pupil (using tropicamide 0.5%) to assess the anterior segment, the lens, and signs of retinopathy (according to the proposed international classification of diabetic retinopathy).^33^ The grading of the lens was made in all cases using the LOCS III system^34^ to exclude the possibility of lens opacification. Altogether, 57 patients (30 diabetic and 27 control individuals) were enrolled in the study.

### Statistical Analysis

Data were expressed as mean ±SD. Thyroid hormone levels and cone numbers in rats were compared using exact two-sample Fisher–Pitman permutation tests. In the case of human demographic data, continuous variables were compared with the Mann–Whitney *U* test and categorical variables with Fisher's exact test without multiplicity correction. The SDs of fT4 levels of control and diabetic human patients were compared with an *F*-test. Logistic regression models were created for CVD as the outcome. The final model included fT4, age, gender, and diabetes as predictors. In the diabetic group, a logistic model of CVD dependence from diabetes duration and HbA1c levels was made, controlling for age and gender. The level of significance was set at *P* < 0.05. Statistical analyses were performed using R software (R Foundation for Statistical Computing, Vienna, Austria). Packages for R 3.6.0. were used. For the Fisher–Pitman permutation tests, the coin (v1.3.0) oneway_test function was used. For other relevant results mentioned, the built-in packages (base, for example) stats were used.^35^

## Results

### Dual Cones and Thyroid Hormone Levels in Diabetic Rat Models

**Figure 1.**
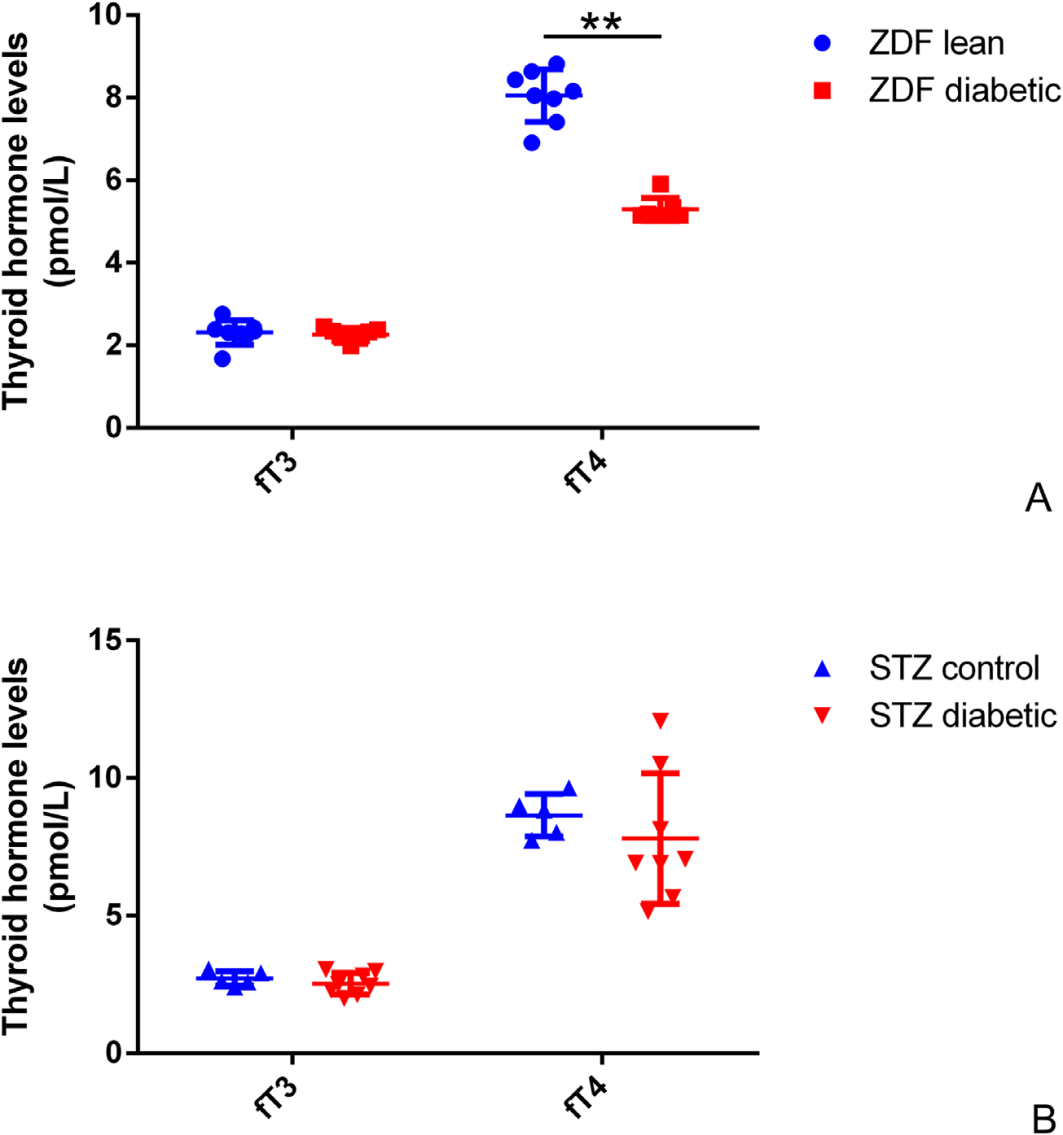
Thyroid hormone levels in ZDF (**A**) and STZ-induced (**B**) diabetic rats compared with controls. Data from all animals used are plotted in the graphs (individual values and mean ± SD for each parameter). *Blue markers* indicate values from controls, and *red markers* indicate values from diabetic specimens. Note that the fT4 levels are significantly lower in ZDF diabetic rats than in ZDF lean controls. In STZ-induced diabetic animals, although the mean fT4 values do not differ from those of the control rats, the SD values are larger compared with controls. ^**^P < 0.001.

S- and M-opsin–positive dual cones were frequently visible in both STZ-induced and ZDF rats. In ZDF rats, only sections were used for evaluation due to the low sample size. Near the ora serrata, all cones were dual in nature in all ZDF diabetic specimens scanned. These results have been published earlier.^14^ Parallel changes were detectable in the thyroid hormone levels (Fig. 1A). Although the fT3 levels were not different between the two groups (2.31 ± 0.3 pmol/L in lean and 2.27 ± 0.16 pmol/L in diabetic rats; *P* = 0.7537), the fT4 levels were significantly lower in diabetic specimens (8.05 ± 0.63 pmol/L in lean vs. 5.29 ± 0.28 pmol/L in diabetic specimens; *P* = 0.0002); however, the actual decrease is underestimated with this method. The fT4 levels were under the detection limit in four out of seven diabetic animals examined. In these cases, the lower limit of detectability was used for the statistics (5.15 pmol/L).

In STZ-induced diabetic rats, both the thyroid hormone levels and the number of dual cones varied remarkably between individual specimens examined. No significant difference was detectable in fT3 levels between control and diabetic specimens (2.72 ± 0.27 pmol/L vs. 2.52 ± 0.39 pmol/L; *P* = 0.3395) (Fig. 1B). Unlike in ZDF rats, the mean fT4 levels were not significantly different in the two groups (8.65 ± 0.77 pmol/L control vs. 7.80 ± 2.37 pmol/L diabetic; *P* = 0.4669). However, fT4 levels in diabetic specimens ranged from 5.15 pmol/L (the lower limit of detectability) to 12.07 pmol/L. This variation did not change the mean value but more than doubled the SD in the diseased group compared to controls.

**Figure 2.**
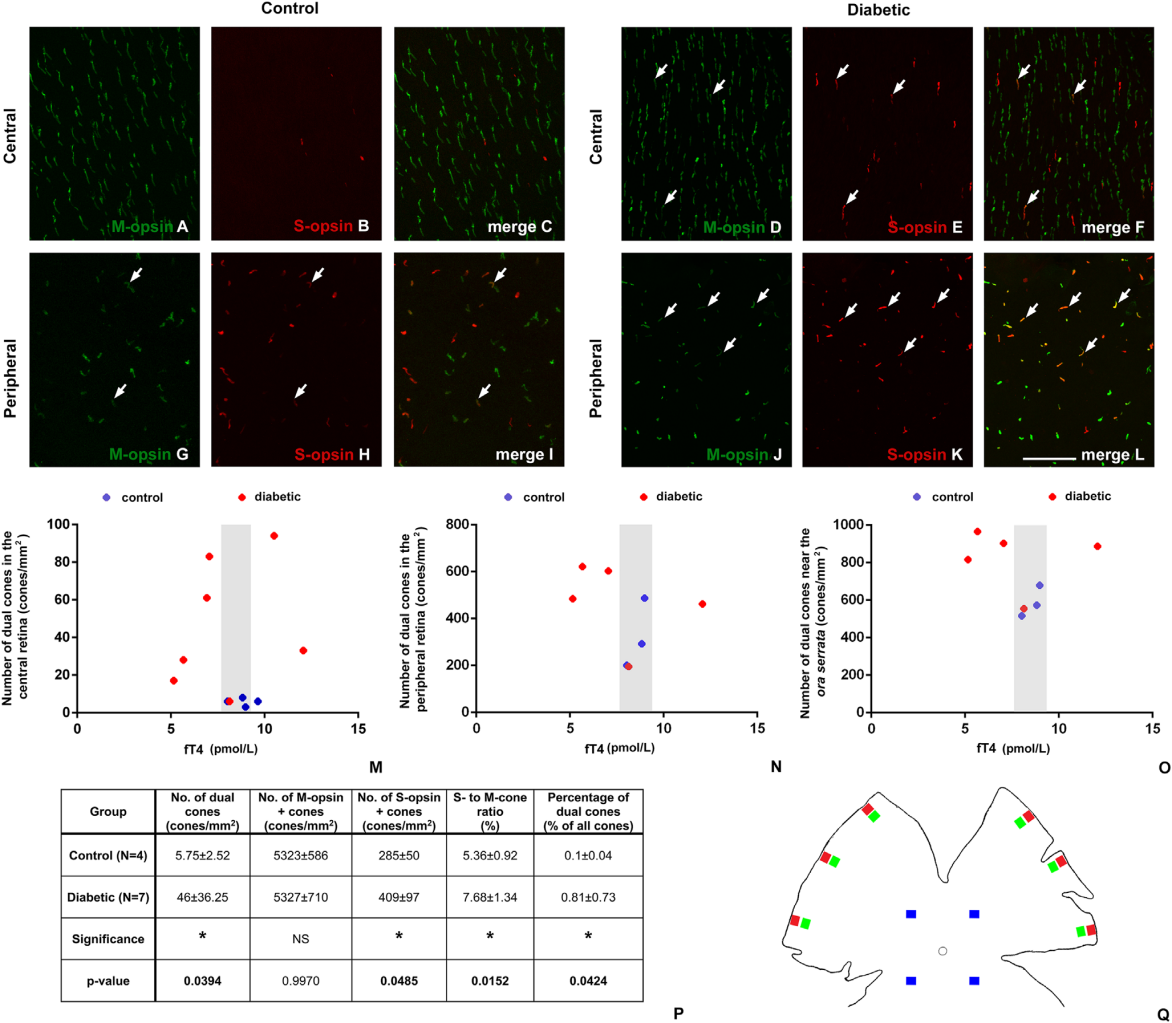
Dual cones and fT4 levels in STZ-induced diabetic rats. Representative images from control (**A**–**C**, **G**–**I**) and diabetic (**D**–**F**, **J**–**L**) rat retinas stained for S-opsins (in *red*) and M-opsins (in *green*). Some dual-stained elements are marked with *arrows*. Note that in diabetic specimens, dual cones are present in higher number than in controls, especially in the peripheral regions. In central retinal regions, dual cones are almost completely missing in control rats and are present only in a relatively small number in diabetes. Figures **M** to **O** show data from three different regions: central (**M**), peripheral (**N**), and extreme peripheral regions (near the ora serrata, **O**). Data of control animals are labeled with *blue markers* and those for diabetic animals with *red markers*. The mean ± SD fT4 level of the controls (8.65 ± 0.77 pmol/L) is indicated with *gray shading*. Results show that the number of dual cones in diabetic rats tends to be higher in case of fT4 levels outside this range. Diabetic animals with fT4 in the mean ± SD range of the controls exhibited dual cone numbers close to control numbers. (**P**) Mean numbers and statistical analysis of S-opsin and M-opsin–positive cones, dual cones, and total cone numbers, as well as the ratio of S-cones to M-cones and the percentage of dual cones, are presented for each group as estimated from the central counting frames. (**Q**) Schematic drawing summarizing the positions of the counting frames for estimating central, peripheral, and extreme peripheral dual cone values (*blue*, *green*, and *red* quadrangles, respectively). ^*^P < 0.05; NS, not significant. *Scale bar*: 20 µm.

In STZ-diabetic retinas, more S- and M-opsin–positive dual cones were detectable than in controls (Fig. 2). With regard to the fT4 levels, the diabetic group contained animals within the mean ± SD range of the controls and also some animals with fT4 levels above or below this range. When dual cone numbers were plotted against fT4 levels, a clear tendency was observed. The numbers tended to be higher in specimens where fT4 levels were either above or under the control range (Figs. 2M–O). This tendency seemed to be valid in all retinal regions examined. Furthermore, in diabetic specimens with fT4 levels within the mean ± SD range of the controls, the number of dual cones was similar to that in the controls.

In addition to dual cone number, data were also collected on the number of S- and M-opsin–positive cones from the central retinal regions in STZ-induced diabetic rats. This allowed us to calculate the ratio of S-cones to M-cones, total cone number, and percentage of dual cones of the total cone population. The results are summarized in Figure 2P. The data certainly exclude any major loss of cone cells due to apoptosis and confirm an increase in the number and percentage of dual cones. The difference was significant in both parameters (*P* = 0.0394 for dual cone numbers and *P* = 0.0424 for percentages). Furthermore, an increase in the number of S-opsin–positive cone numbers (*P* = 0.0485), as well as in the ratio of S-cones to M-cones (*P* = 0.0152), was also detectable.

### Dual Cones in Postmortem Human Retinal Samples

**Figure 3.**
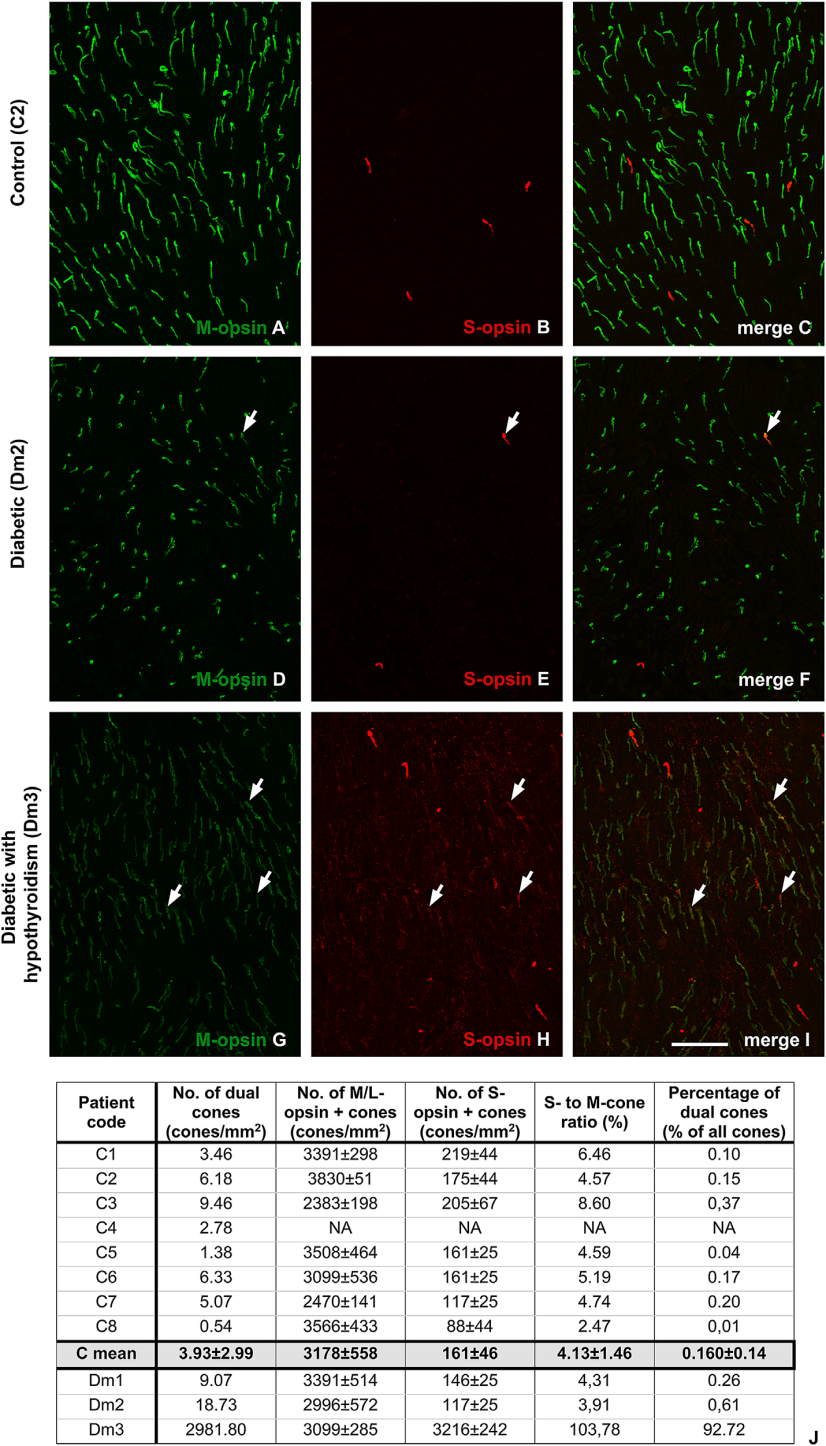
Dual cones in human peripheral retinal samples. Samples were analyzed using S-opsin (in *red*) and M-opsin (in *green*) specific antibodies. Images were taken from peripheral retinal regions, immediately next to the ora serrata. In non-diabetic donors (**A**–**C**), dual cones were present only in small numbers, but they were frequently visible in diabetic retinas (**D**–**I**; some dual cones are marked by *arrows*). Furthermore, in one donor with diabetes and hypothyroidism (the patient identified as Dm3; see Table 1 for details), almost all cones were dual in nature (**G**–**I**). The results of quantification including the mean numbers of S-, M-/L-opsin–positive cones, dual cones, and total cone numbers, as well as the ratio of S-cones to M-cones and the percentage of dual cones, are presented for each retina (**J**). Mean values for the control group were also calculated. *Scale bar*: 50 µm.

There were at least twice as many S- and M/L-opsin–positive dual cones in postmortem diabetic human retinas as in non-diabetics, counted in identical areas (Fig. 3). In one case (in donor Dm3, who had diabetes and documented hypothyroidism), almost all cones were dual in the region examined (Figs. 3G–I).

Quantified data for each donor are shown in Figure 3J. In addition to an increase in dual cone numbers in diabetes, no major change or evident trend was detectable in the S- to M/L-cone ratio or in cone numbers.

### Case–Control Study: Visual Function and Thyroid Hormone Levels

Clinical characteristics of control and diabetic patients are summarized in Table 2.

**Table 2.** Summary of Data Collected From the Patients of the Control and Diabetic Groups With Statistical Analysis

	Control Group						Diabetic Group						Statistics	
Continuous Variables	Valid *N*	Mean	SD	Q25	Median	Q75	Valid *N*	Mean	SD	Q25	Median	Q75	Significance	*P*
Age (d)	27	20,245.04	2691.81	18,828.00	20,335.00	21,845.00	30	20,835.57	3545.02	18,468.00	22,096.50	23,457.00	NS	0.2706
Weight (kg)	26	84.08	15.51	70.00	82.50	92.00	30	85.02	16.95	70.00	83.65	100.00	NS	0.9803
Height (cm)	26	170.50	9.98	164.00	170.00	179.00	30	168.90	10.72	162.00	168.00	176.00	NS	0.5211
BMI (kg/m^2^)	26	29.38	5.62	24.66	28.04	31.83	30	29.70	4.697	26.04	29.04	32.42	NS	0.6873
Fasting blood glucose (mmol/L)	27	5.12	0.46	4.80	5.20	5.50	23	8.07	2.410	6.20	7.40	10.30	—^***^	**<0.0001**
TSH (mU/L)	27	1.44	0.59	1.29	1.29	1.83	30	1.33	0.53	0.90	1.32	1.72	NS	0.6173
fT3 (pmol/L)	27	4.28	0.678	3.78	4.30	4.69	30	4.15	0.49	3.82	4.11	4.50	NS	0.2306
fT4 (pmol/L)	27	12.72	1.341	11.72	12.73	13.89	30	12.62	1.971	11.10	12.48	14.05	NS	0.7013
**Categorical Variables**	**Outcome 1 (*n*/Valid *N*)**			**Outcome 2 (*n*/Valid *N*)**			**Outcome 1 (*n*/Valid *N*)**			**Outcome 2 (*n*/Valid *N*)**			**Significance**	***P***
Gender	M (15/27)			F (12/27)			M (17/30)			F (13/30)			NS	1.000
CVD	Y (2/27)			N (25/27)			Y (11/30)			N (19/30)			—^*^	**0.0114**
Hypertension	Y (17/27)			N (10/27)			Y (23/30)			N (7/30)			NS	0.3851
Hyperlipidemia	Y (11/27)			N (16/27)			Y (24/30)			N (6/30)			—^**^	**0.0031**
Smoking	Y (8/27)			N (19/27)			Y (8/30)			N (22/30)			NS	1.000
Alcohol	Y (2/27)			N (25/27)			Y (2/30)			N (28/30)			NS	1.000

Continuous variables were compared by Mann–Whitney U tests and categorical variables with Fisher's exact tests without multiplicity correction. *P* < 0.05 was considered statistically significant. ^*^P < 0.05; ^**^P < 0.01; ^***^P < 0.001; NS, not significant. BMI, body mass index; FBG, fasting blood glucose; Y, the categorical variable is present in the population; N, the categorical variable is absent from the population.

There was no significant difference between the diabetic and the control groups with regard to age, gender, body mass index, blood pressure, alcohol consumption, or smoking habits. Fasting blood glucose levels were normal in the control group, excluding the possibility of latent diabetes. Diabetic patients had HbA1c levels between 5.3 and 10 mmol/L. The percentage of hyperlipidemic patients was higher in the diabetic group than in the control group.

With the Lanthony test, a higher occurrence of manifest CVDs was registered in the diabetic group (7.0% control vs. 36.7% diabetic; *P* = 0.011). The dominant type of color vision deficiency was tritan; however, the protan axis was also affected in some cases. Six diabetic patients had tritan CVDs and two patients were making errors on the protan axis. In three patients, no axis was detectable. In the control group, one patient made tritan-like errors and another made deutan-like errors. Lens grading was performed in all patients. All diabetic and control subjects’ lenses were below or equal to N1, C1, P0.

Serum TSH, fT3, and fT4 levels were all within the normal range in all patients. There was no significant difference in the average TSH and fT3 levels between control and diabetic patients, and the SDs were also comparable. There was no significant difference in the average fT4 values, either; however, a tendency for increased SD was visible in diabetic patients (1.341 pmol/L control vs. 1.971 pmol/L diabetic), similar, albeit not as prominent, as what was observed in the STZ-induced rat model. Statistical analysis with the *F*-test revealed a *P* value of 0.0505; thus, this difference in the SD values between control and diabetic subjects was not significant. We also compared the data of all patients in the Manifest CVD subgroups (from both the control and diabetic groups) with the No CVD subgroups (Table 3). A significant difference was detectable only in the percentage of diabetes and in the average age of the patients. Serum TSH, fT3, and fT4 levels did not correlate with the presence of CVDs.

**Table 3.** Statistical Analysis of the Data Collected From All Patients With CVDs and Without CVDs

	No CVD Group						Manifest CVD Group						Statistics	
Continuous Variables	Valid *N*	Mean	SD	Q25	Median	Q75	Valid *N*	Mean	SD	Q25	Median	Q75	Significance	*P*
Age (d)	44	19,899.43	3109.93	17,118.00	20,011.00	22,395.50	13	22,815.31	2101.54	21,689.00	22,850.00	23,561.00	—^**^	**0.0029**
Weight (kg)	43	86.01	15.69	73.50	84.30	100.00	13	79.86	17.42	65.00	80.00	93.00	NS	0.2515
Height (cm)	43	170.86	10.19	164.00	168.00	179.50	13	165.62	10.09	156.00	164.00	174.00	NS	0.1154
BMI (kg/m^2^)	43	29.71	5.01	26.13	28.69	31.72	13	29.06	5.57	25.76	27.12	32.51	NS	0.6344
Fasting blood glucose (mmol/L)	41	6.41	2.18	5.20	5.60	6.60	9	6.77	2.53	5.20	6.00	7.20	NS	0.4866
TSH (mU/L)	44	1.37	0.56	0.95	1.29	1.70	13	1.44	0.54	1.10	1.53	1.77	NS	0.6051
fT3 (pmol/L)	44	4.23	0.52	3.86	4.25	4.61	13	4.13	0.77	3.82	4.11	4.78	NS	0.9848
fT4 (pmol/L)	44	12.62	1.51	11.31	12.70	13.81	13	12.81	2.26	10.96	12.78	14.48	NS	0.9242
**Categorical Variables**	**Outcome 1 (*n*/Valid *N*)**			**Outcome 2 (*n*/Valid *N*)**			**Outcome 1 (*n*/Valid *N*)**			**Outcome 2 (*n*/Valid *N*)**			**Significance**	***P***
Gender	M (26/44)			F (18/44)			M (6/13)			F (7/13)			NS	0.5283
Diabetes	Y (19/44)			N (25/44)			Y (11/13)			N (2/13)			—^*^	**0.0114**
Hypertension	Y (29/44)			N (15/44)			Y (11/13)			N (2/13)			NS	0.3044
Hyperlipidemia	Y (25/44)			N (19/44)			Y (10/13)			N (3/13)			NS	0.3310
Smoking	Y (11/44)			N (33/44)			Y (5/13)			N (8/13)			NS	0.4831
Alcohol	Y (3/44)			N (41/44)			Y (1/13)			N (12/13)			NS	1

Color vision tests were completed by all participants of the study. Data collected from subjects with CVDs were compared with those obtained from participants without manifest CVD. In the two groups, only the average age of the patients and the number of patients with diabetes differed significantly. Continuous variables were compared by Mann–Whitney *U* tests and categorical variables with Fisher's exact tests without multiplicity correction. *P* < 0.05 was considered statistically significant. ^*^P < 0.05; ^**^P < 0.01.

A logistic regression model was created for CVD as the outcome, including fT4, age, gender, and diabetes as predictors (Table 4A). The model confirmed the major role of age in the development of CVDs (*P* = 0.0152). Furthermore, it confirmed that diabetes also contributes significantly to the development of color vision alterations (*P* = 0.0319) but failed to indicate the possible role of fT4 in the development of CVDs. The details of the analysis are given in Table 4A.

**Table 4.** Probability of CVDs When Considering Some of the Most Important Risk Factors

A. Results of the Logistic Regression Model for CVD As An Outcome in All Patients		
Predictor	*P*	Odds Ratio 95% CI (Manifest CVD vs. No CVD)
Diabetes	**0.0319**	**1.36–49.55** (for diabetic)
Age	**0.0152**	**1.05–1.38** (1 y)
Gender	0.2452	0.55–14.51 (female)
fT4	0.5207	0.55–1.34 (1 pmol/L)
**B. Results of the Logistic Regression Model for HbA1C Dependency of CVD in the Diabetic Group Controlled for Age and Gender**		
HbA1C	0.9130	0.38–2.41 (1%)
Age	**0.0410**	**1.02–1.32** (1 y)
Gender	0.3664	0.40–14.4 (female)
**C: Results of the Logistic Regression Model for the CVD Dependency on the Duration of Diabetes in the Diabetic Group, Controlled for Age and Gender**		
Duration of diabetes	0.2064	0.59–1.09 (1 y)
Age	**0.0315**	**1.03–1.35** (1 y)
Gender	0.2593	0.49–18.56 (female)

In the clinical study, we estimated the dependence of CVDs from some of the most important risk factors using logistic regression models. The three different models represent CVD probability in the whole study population (A) and in diabetic patients only (B and C). From all risk factors, only age and diabetes were associated with CVDs. In the population examined, diabetes control (HbA1C, B) and disease duration (C) did not seem to increase CVD probability significantly. CI, confidence interval.

Comparisons were also made between diabetic patients in the Manifest CVD and No CVD subgroups. Data are presented in Table 5. No statistical analysis was performed due to the small number of cases. Logistic models of CVD dependence from diabetes duration and glycemic control (HbA1C levels) were performed (controlling for age and gender); however, they did not show a significant difference (Tables 4B, 4C).

**Table 5.** Summary of Data Collected From Diabetic Patients With and Without Color Vision Deficiency

	Diabetic Patients in the No CVD Group						Diabetic Patients in the Manifest CVD Group					
Continuous Variables	Valid *N*	Mean	SD	Q25	Median	Q75	Valid *N*	Mean	SD	Q25	Median	Q75
Age (d)	19	19,729.42	3731.35	16,498.00	19,504.00	22959.00	11	22,746.18	2245.73	20,437.00	22,847.00	23,558.00
Weight (kg)	19	88.13	17.725	72.00	85.00	105.00	11	79.65	14.763	65.00	80.00	93.00
Height (cm)	19	170.58	10.849	164.00	168.00	180.00	11	166.00	10.325	156.00	164.00	176.00
BMI (kg/m^2^)	19	30.12	4.459	26.22	29.40	32.41	11	28.98	5.223	25.76	27.12	32.51
Fasting blood glucose (mmol/L)	16	8.41	2.314	6.35	7.55	10.75	7	7.31	2.636	5.70	6.60	7.50
HbA1c (%)	19	6.72	0.688	6.10	6.90	7.40	11	7.03	1.477	5.70	7.00	7.50
TSH (mU/L)	19	1.33	0.574	0.86	1.31	1.68	11	1.33	0.480	0.98	1.32	1.77
fT3 (pmol/L)	19	4.10	0.494	3.59	4.09	4.50	11	4.22	0.491	3.82	4.55	4.78
fT4 (pmol/L)	19	12.61	1.768	11.12	11.12	14.05	11	12.63	2.375	10.83	14.22	14.48
Duration of diabetes (y)	19	5.11	3.526	2.00	5.00	9.00	11	5.27	3.165	2.00	5.00	8.00
**Categorical Variables**	**Outcome 1 (*n*/Valid *N*)**			**Outcome 2 (*n*/Valid *N*)**			**Outcome 1 (*n*/Valid *N*)**			**Outcome 2 (*n*/Valid *N*)**		
Gender	M (11/19)			F (8/19)			M (6/11)			F (5/11)		
Hypertension	Y (14/19)			N (5/19)			Y (9/11)			N (2/11)		
Hyperlipidemia	Y (15/19)			N (4/19)			Y (9/11)			N (2/11)		
Smoking	Y (18/19)			N (1/19)			Y (4/11)			N (7/11)		
Alcohol	Y (2/19)			N (17/19)			Y (0/11)			N (11/11)		

Diabetic patients were enrolled into these groups based on the results of the Lanthony color vision test. Due to the low number of patients in the diabetic subgroups, no detailed statistical analysis was performed.

**Figure 4.**
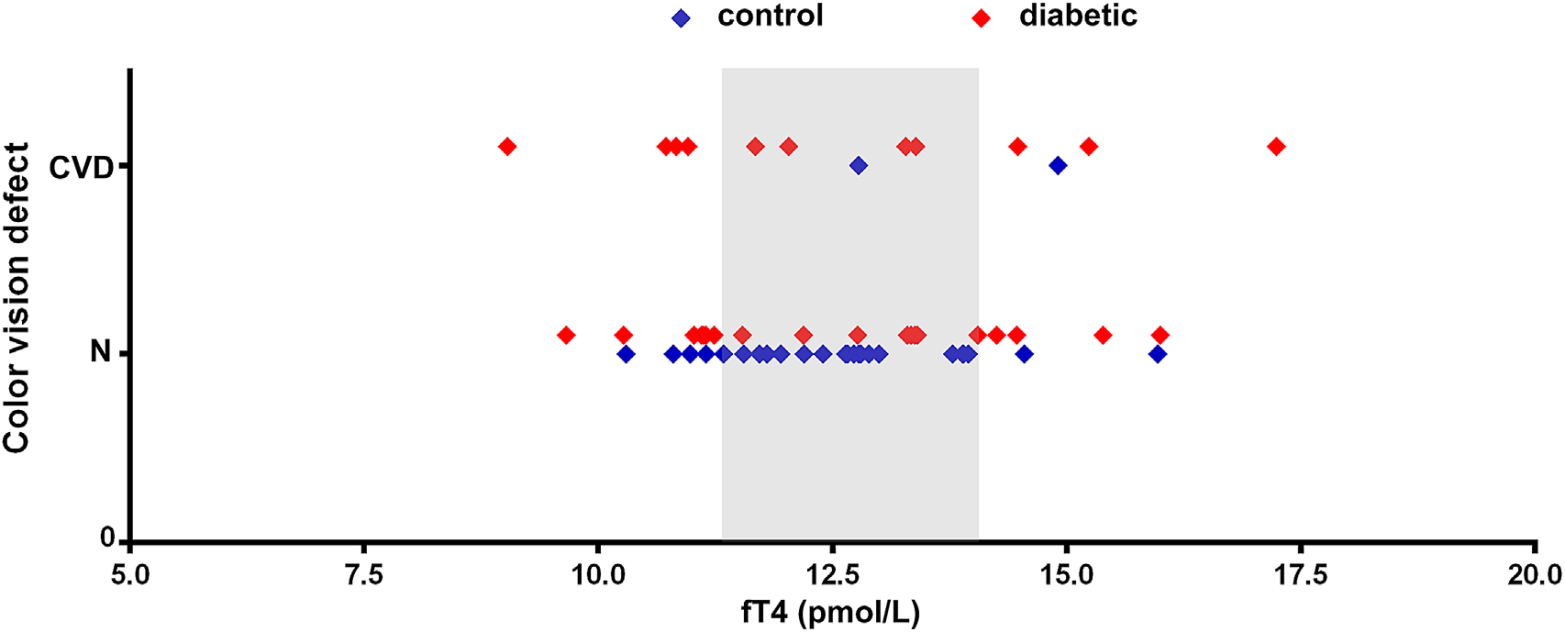
Color vision and fT4 levels in human diabetic and control patients. The fT4 levels are plotted against color vision capacity for each individual patient participating in the study. All fT4 data are in the normal range. *Blue markers* label non-diabetic participants (*n* = 27); *red markers* label diabetic patients (*n* = 30). For comparison with animal data, *gray shading* highlights the mean ± SD range of the controls for fT4 level (12.72 ± 1.34 pmol/L) of the control group of patients. Note that, in cases of higher or lower levels, color vision deficiencies tend to occur more often in the entire population examined (but not in the diabetic subgroup). N, patient with normal color vision.

In rats, dual cone numbers tended to be higher in the case of fT4 levels above or below the mean ± SD range of the controls. Therefore, in the human case–control study, we tried to assess whether fT4 levels above or below the mean ± SD range of the controls enhanced the risk of CVDs. We plotted the data of all patients against fT4 levels and applied the same classification as in the case of the rats (Fig. 4). Data below or above the mean ± SD range of the controls were considered low or high, respectively. The chance for CVDs with fT4 levels falling within the mean ± SD range of the controls was 17.25% for all patients, compared with 26.66% for low and 40% for high fT4 levels. In the diabetic group alone, the three ranges were equally represented (40% of diabetic patients with CVDs had fT4 levels in the mean ± SD range of controls, 36.36% were in the low range, and 23.33% were in the high range). That is, even though CVDs tend to occur at both higher and lower fT4 levels in the total examined population, the correlation was clearly not present in the diabetic patient group.

## Discussion

The fact that certain visual functional alterations are common among subjects with no clinically apparent diabetic retinopathy has been published earlier.^5^^,^^8^^,^^36^^,^^37^ Although in terms of quality of life the burden of functional deficits with normal vision (such as CVDs or decreased contrast sensitivity) seems to be minor, these may still be important considering their potential contribution to or prognostic value for the development of vasculopathy. Color vision tests are simple, available, non-invasive tools that can be used to screen visual function and diabetes-related damage of photoreceptors, which may be among the first manifest signs of neurodegeneration. Furthermore, photoreceptor damage may initiate the vascular alterations leading to advanced retinopathy. Due to diabetes, the dysregulated metabolic environment of the photoreceptors and retinal pigment epithelium^12^^,^^14^^,^^38^ leads to increased oxidative stress and local inflammatory changes, with consequent development of vascular lesions causing vascular leakage with macular edema and later leading to neovascularization. Additionally, photoreceptors release pro-inflammatory factors (such as COX2, ICAM-1, and iNOS), contributing to damage of the nearby endothelial cells.^10^^,^^11^

Discrimination of color vision may be influenced by several ophthalmological parameters (such as cataract formation of the lens and increased intraocular pressure) and metabolic factors, including dyslipidemia and elevated blood pressure.^6^^,^^8^ The precise pathogenesis of CVDs in early diabetes is still not fully understood. We demonstrated earlier an increase in the number of dual cones—cones coexpressing S- and M-opsins simultaneously—in diabetic rat retinas.^12^^,^^14^ Electrophysiologic examinations in murine retinas indicate that the presence of two photopigments in cones diminishes color selectivity even if one of the opsins is present in a very small quantity.^20^ Although no functional data are available from human patients, it is logical to assume that an increase in dual cone numbers, if it can be confirmed in diabetes, may contribute to the CVDs detectable in diabetic patients.

As normal thyroid homeostasis is an essential factor for normal cone opsin expression and photoreceptor viability,^31^^,^^39^ we tested here whether thyroid hormonal status was related to the increased number of dual cones and as a possible consequence of CVDs detectable in diabetic patients, as well. In the present work, we observed an increased number of dual cones in type 1 and type 2 diabetic rat models and in the human diabetic retina and that the number of dual cones seemed to correlate with fT4 levels in both rat models, albeit not as originally proposed, as both lower and higher fT4 levels correlated positively with an increase in dual cone numbers. The pilot case–control clinical study, however, failed to prove the suspected correlation between thyroid hormone levels and the probability of CVDs.

### Opsin Expression Pattern in Diabetes

Although the potential pathological alterations of photoreceptors in diabetes have been proposed and documented by many reports,^40^^–^^42^ the switch in opsin expression pattern is a relatively new discovery. Our workgroup has previously demonstrated an evident increase in the number of dual cones in STZ-induced diabetic rat models 12 weeks after diabetes induction,^12^ as well as in 6-month-old ZDF rats.^14^ Furthermore, the increase in S-cone numbers was also detected in *Meriones shawi*, a recently described model of metabolic syndrome and diabetes, suggesting that a similar process is in progress here, as well,^43^ indicating the robustness of this effect. The results presented here from rats 8 weeks after diabetes induction with STZ fit well into this scheme, demonstrating that dual elements appear even earlier in the course of the disease. The number of dual cones detected was slightly higher than that after 12 weeks of diabetes.^12^ This may be due to the duration of the disease or, more probably, to the more sensitive immunohistochemical procedure used in this experiment, confirming previous observations that dual cone numbers detected depend on the sensitivity of the detection system used.^44^

Interestingly, when examining cone numbers from the central retinal regions of STZ-induced diabetic rats, a significant increase in S-cone numbers as well as ratios of S-cones to M-cones in diabetic specimens were noted. This may be explained by the proposition that some dual cones might have already transformed to exclusively or near exclusively S-opsin–containing cones, similarly to what was suggested in the case of *Meriones shawi.*^43^ This would also indicate that the actual number of cones undergoing transition can be even higher than originally assumed from dual cone data alone. Due to the low number of cases and the high SD values detected, this possibility certainly requires further confirmation.

In humans, the number of S- and M/L-opsin–positive dual cones in diabetes has not been previously examined. Data from non-diabetic postmortem human retinas are available from the central retinal regions only,^45^ where estimated numbers fell in the range of 0.01% to 0.03%. This is almost an order of magnitude lower than what we detected at the ora serrata in donors of the control group; the number was even higher in the diabetic donors. We managed to demonstrate that the human retina behaves similarly to those of the often-used diabetes model animals, at least in this aspect. However, we must point out that the change in humans is less prominent than in rats (except for donor Dm3, where practically all cones were dual in nature), which raises questions about the functional consequences of this discovery.

### Thyroid Hormone Levels and Cone Opsin Expression in Diabetes

Thyroid disorders and the pathogenesis of diabetes are associated through complex biochemical, genetic, and hormonal pathways. There are common pathways in the pathophysiology of type 2 diabetes and thyroid malfunctions (e.g., metabolic factors, oxidative stress). Also, in type 1 diabetes, insulin and thyroid hormones may both be affected by autoimmunity.^26^^,^^28^^,^^46^ The prevalence of thyroid disorders (including subclinical hypo- and hyperthyroidism) is two to three times higher in the diabetic population.^26^^,^^47^ Pathological and subclinical thyroid changes in diabetes increase the prevalence and progression of diabetic vascular complications, including retinopathy.^48^

It is also known that normal thyroid homeostasis is essential in the development and determination of opsin expression and cone viability in both rodent and human retinas.^31^^,^^39^^,^^49^^,^^50^ The published data suggest that thyroid hormones are also necessary in maintaining cone opsin identity in adults, although there is some uncertainty. In one report,^51^ induced hypothyroidism had no effect on cone opsin expression in adult mice after 2 weeks, whereas Glaschke et al.^24^ reported changes in the opsin expression pattern first at 5 to 7 weeks after serum thyroid hormones decreased to hypothyroid levels, concluding that the process may be influenced by the duration of hypothyroidism. Furthermore, experiments on *Fukomys anselli* (with naturally low serum thyroxine levels and S-opsin–dominated retinas) suggest an age dependence; in young animals, T4 supplementation caused an increase in M-opsin expression levels, whereas in older animals it failed to produce such an effect.^52^

Our results also present evidence that thyroid hormones are necessary to maintain opsin expression patterns in adult rats. Consistent with another study on STZ-diabetic rats, only fT4 and not fT3 levels showed changes in the present study.^53^ Furthermore, interestingly and unexpectedly, the connection between fT4 levels and dual cone numbers was not monotonic as originally assumed. Instead, our results indicate that the number of dual cones in the diabetic rat retina increases in the case of both higher and lower fT4 levels than the control average. Unfortunately, this observable trend could not be tested statistically because the statistical validation of such a non-monotonic relationship would have required a much greater number of specimens. The duration of the fT4 level alteration would also have to be taken into account in such an evaluation. At present, the precise mechanism by which higher fT4 levels may alter opsin expression is unknown, calling for further research. It may be related to the fact that an excess of thyroid hormones, acting through β2 receptors, is toxic for cones but not rods during development.^31^

Furthermore, it is possible that other factors not addressed in the current study could also contribute to the presence of dual cones in diabetes. For example, the phagocytotic activity of retinal pigment epithelial cells has been shown to be reduced in this disease.^54^ Therefore, the removal of the photopigments from the outer segment of cone cells (renewal of outer segments) is slower in diabetes, which may also impact the detectability of dual cones.

A similar connection between opsin expression and thyroid hormone levels is assumed for the human retina, as well. Thyroid hormone receptor β2 was proven to control cone development,^49^ and decreased color contrast sensitivity was reported in hypothyroid patients,^55^ as well as in preterm infants with reduced postnatal thyroid hormone levels.^56^ Interestingly, as already mentioned, in our study the retina of a diabetic subject with hypothyroidism (donor Dm3) showed practically all cones in the peripheral retina co-expressing cone photopigments.

### Thyroid Hormone Levels and Color Vision

In light of these data, we conducted a pilot study to examine the effect of thyroid hormone levels on color vision in human patients. To the best of our knowledge, this is the first study analyzing the role of thyroid hormonal status in the development of human diabetes-related retinal alterations. In agreement with other reports,^8^^,^^36^^,^^37^^,^^57^ we found a significantly larger occurrence of CVDs without manifest retinopathy in the diabetic group than in the control group. Published data suggest that, besides age and gender, metabolic factors including body mass index, blood pressure, lipid profile, duration of disease, and patient compliance (assessed by the degree of diabetic control) all affect the risk of CVDs in diabetes.^6^^,^^8^ We correlated these with the occurrence of CVDs. Aside from age, no significant difference was found in our study population. The age ranges of the healthy group and diabetic group were not significantly different, although CVDs appeared in the elderly range, in both the overall population and the diabetic subgroup. (Patients with CVDs in the entire assessed population were around 8 years older than the non-CVD patients.) The logistic regression models also confirmed the major effect of both age and diabetes in the development of CVDs in the overall population. Interestingly, contrary to earlier data,^6^^,^^8^ neither duration nor the control of diabetes seemed to influence the development of CVDs in our diabetic subgroup. The effect of age was robust in the study, despite all subjects’ lenses being graded below or equal to N1, C1, P0 with the LOCS III system grading; thus, lens yellowing was not detectable in any of our patients. However, aging and duration of diabetes both influence the lens center thickness and lens density,^54^ which may, in turn, have been responsible for the increased risk of CVDs in our older patients. The dominant axis of CVDs with a Lanthony test was tritan, although protan and no-axis deficiencies also occurred in accordance with other reports.^58^ With the present methods used (Lanthony and Ishihara), the cause of CVDs outside the tritan axis remains unexplained, although the possibility of congenital deficiencies cannot be ruled out. Interestingly, a study of hypothyroid patients also reported errors on both tritan and protan axes, all of which showed significant improvement with normalizing thyroid status,^55^ indicating that thyroid hormones may be connected to protan anomalies, as well. This fact also warrants further studies in diabetic patients.

Thyroid hormone assessment did not reveal any thyroid disease in our patients in any of the groups, and there was also no significant difference in mean hormone levels. Only a tendency of increased SDs of fT4 levels in diabetic patients was observed, similar to the data for STZ-induced diabetic rats, but this change was not significant. When we examined the possibility that fT4 levels below or above the range of the mean ± SD values of the controls may increase the risk of CVDs in human patients, a higher risk was detectable when analyzed in the complete study population but not in the diabetic group alone. To summarize, despite some slight similarity to the animal models, these data do not support our original view that the development of CVDs is a thyroid-dependent process in diabetic subjects. The current results may be attributed to the relatively small number of patients involved and to the high genetic heterogeneity of human patients compared with inbred animal models. Furthermore, we must point out that all diabetic and control subjects in the present study were clinically euthyroid. It remains a challenging question for further study how much these visual functions are affected in manifest hypo- or hyperthyroid diabetic subjects.

Certain limitations of our case–control study must be mentioned. First, it was a single time point evaluation of retinopathy and thyroid hormones, with a relatively low number of participants. Additionally, more advanced color vision tests and different in vivo, non-invasive and invasive retinal imaging techniques may be required to properly reveal the role of thyroid hormones in the development of CVDs.

As it is certain that opsin expression changes in diabetes, the clinical aspects of our observation require further studies with a larger number of patients. We hope that our preliminary results serve as a good basis to design such a study. The literature suggests that controlling blood glucose levels alone may not be sufficient to prevent the development of diabetic retinopathy, and other concomitant changes—including altered hormone levels—must also be given more attention in the future.^28^ The further assessment of our theory that thyroid hormone levels could serve as possible early markers of retinopathy and photoreceptor involvement may help to clarify their possible clinical and therapeutic role in the prevention of diabetes-related visual complications in human patients.
